# RIG-I, a novel DAMPs sensor for myoglobin activates NF-κB/caspase-3 signaling in CS-AKI model

**DOI:** 10.1186/s40779-021-00333-4

**Published:** 2021-06-21

**Authors:** Peng-Tao Wang, Ning Li, Xin-Yue Wang, Jia-Le Chen, Chen-Hao Geng, Zi-Quan Liu, Hao-Jun Fan, Qi Lv, Shi-Ke Hou, Yan-Hua Gong

**Affiliations:** 1 General Hospital of Tianjin Medical University, Tianjin, 300052 China; 2 Institute of Disaster Medicine, Tianjin University, Tianjin, 300072 China; 3 State Key Laboratory of Medicinal Chemical Biology, Nankai University, Tianjin, 300350 China; 4 Tianjin Key Laboratory of Disaster Medicine Technology, Tianjin, 300072 China; 5 Wenzhou Safety (Emergency) Institute, Tianjin University, Wenzhou, 325000 China

**Keywords:** Crush syndrome, Acute kidney injury, Retinoic acid-inducible gene I, Myoglobin, Nuclear factor kappa-B/caspase-3, Damage-associated molecular patterns

## Abstract

**Background:**

Acute kidney injury (AKI) is the main life-threatening complication of crush syndrome (CS), and myoglobin is accepted as the main pathogenic factor. The pattern recognition receptor retinoicacid-inducible gene I (RIG-I) has been reported to exert anti-viral effects function in the innate immune response. However, it is not clear whether RIG-I plays a role in CS-AKI. The present research was carried out to explore the role of RIG-I in CS-AKI.

**Methods:**

Sprague-Dawley rats were randomly divided into two groups: the sham and CS groups (*n* = 12). After administration of anesthesia, the double hind limbs of rats in the CS group were put under a pressure of 3 kg for 16 h to mimic crush conditions. The rats in both groups were denied access to food and water. Rats were sacrificed at 12 h or 36 h after pressure was relieved. The successful establishment of the CS-AKI model was confirmed by serum biochemical analysis and renal histological examination. In addition, RNA sequencing was performed on rat kidney tissue to identify molecular pathways involved in CS-AKI. Furthermore, NRK-52E cells were treated with 200 μmol/L ferrous myoglobin to mimic CS-AKI at the cellular level. The cells and cell supernatant samples were collected at 6 h or 24 h. Small interfering RNAs (siRNA) was used to knock down RIG-I expression. The relative expression levels of molecules involved in the RIG-I pathway in rat kidney or cells samples were measured by quantitative Real-time PCR (qPCR), Western blotting analysis, and immunohistochemistry (IHC) staining. Tumor necrosis factor-α (TNF-α) was detected by ELISA. Co-Immunoprecipitation (Co-IP) assays were used to detect the interaction between RIG-I and myoglobin.

**Results:**

RNA sequencing of CS-AKI rat kidney tissue revealed that the different expression of RIG-I signaling pathway. qPCR, Western blotting, and IHC assays showed that RIG-I, nuclear factor kappa-B (NF-κB) P65, p-P65, and the apoptotic marker caspase-3 and cleaved caspase-3 were up-regulated in the CS group (*P* < 0.05). However, the levels of interferon regulatory factor 3 (IRF3), p-IRF3 and the antiviral factor interferon-beta (IFN-β) showed no significant changes between the sham and CS groups. Co-IP assays showed the interaction between RIG-I and myoglobin in the kidneys of the CS group. Depletion of RIG-I could alleviate the myoglobin induced expression of apoptosis-associated molecules via the NF-κB/caspase-3 axis.

**Conclusion:**

RIG-I is a novel damage-associated molecular patterns (DAMPs) sensor for myoglobin and participates in the NF-κB/caspase-3 signaling pathway in CS-AKI. In the development of CS-AKI, specific intervention in the RIG-I pathway might be a potential therapeutic strategy for CS-AKI.

**Supplementary Information:**

The online version contains supplementary material available at 10.1186/s40779-021-00333-4.

## Background

Crush syndrome (CS), also known as traumatic rhabdomyolysis syndrome, is an acute clinical syndrome characterized by acute kidney injury (AKI), hyperkalemia, myoglobinuria, hypovolemic shock, etc., and is caused by the release of muscle breakdown products into the blood circulation after prolonged compression of skeletal muscles [1, 2]. It often occurs in natural disasters such as earthquakes, tsunamis, and mudslides and human-made disasters such as wars and terrorism [3, 4]. In modern warfare, suddenly produced ruins can lead to large numbers of CS patients. For example, the incidence rates of CS and AKI were 8.2 and 3.3%, respectively, and 41.6% of patients with CS developed AKI after the Wenchuan earthquake in China in 2008 [5]. Therefore, AKI is a main life-threatening complication of CS, and CS-AKI has high morbidity and mortality. At present, myoglobin is well accepted as the main pathogenic factor, as it exerts direct toxic effects and causes tubular obstruction in the kidney [2, 6]. Fluid resuscitation and hemodialysis are the most effective strategies to prevent and treat CS-AKI [7, 8], but they are merely symptomatic treatments. The pathogenesis of CS-AKI is not entirely clear.

Retinoic acid-inducible gene I (RIG-I, also known as RIG-1 or Ddx58) serves as a cytoplasmic RNA sensor and is one of the most important RIG-I-like receptors (RLRs) that regulate innate immunity via recognition of pathogen-associated molecular patterns [9–11]. Upon recognition of various viral RNAs (mainly double-stranded RNA), the RIG-I signaling pathway triggers multiple signaling cascades and induces the overexpression of type I interferons (IFNs) and inflammatory cytokines, which are also generated in various inflammatory diseases such as sepsis, hepatocellular carcinoma and chronic kidney disease [12–14]. The association of RIG-I with traumatic diseases such as irradiation- and immune-mediated gut injury, liver ischemia/reperfusion injury, and spinal cord injury has been reported [15–17]. However, the precise mechanisms underlying this association have not yet been clearly elucidated, especially in CS-AKI. Investigating the relationship between the RIG-I signaling pathway and CS-AKI might provide a molecular target for CS-AKI treatment.

Thus, in the present study, we tested the hypothesis that RIG-I serves as a damage-associated molecular patterns (DAMPs) sensor, recognizing myoglobin and activating the NF-κB/caspase-3 signaling pathway in the development of CS-AKI.

## Methods

### Ethics statement

The experiments involving animals were approved by the Animal Care and Use Ethical Committee of General Hospital of Tianjin Medical University (IRB2021-DW-19) and complied with the Guide for the Care and Use of Laboratory Animals approved by the National Institutes of Health.

### Animals and experimental design

A total of 24 male Sprague-Dawley rats weighing 180–200 g were maintained under standard laboratory conditions (constant temperature of 25 °C and humidity at 50–60% with a 12 h light and 12 h dark cycle) and had free access to food and water. The CS-AKI model was prepared using a crush injury platform [18, 19]. The rats were randomly divided into two groups, including the sham group and the CS group (*n* = 12). After administration of anesthesia, the double hind limbs of rats in the CS group were put under a pressure of 3 kg for 16 h. The rats in both groups were denied access to food and water. After relieving the pressure, all rats were allowed free access to food and water, and no deaths events were observed. Rats in each group were sacrificed at 12 h or 36 h (*n* = 6) after pressure was relieved. Blood was extracted by aorta ventralis puncture. The kidney tissue was quickly removed and stored until quantitative real-time PCR (qPCR), Western blotting, and immunohistochemistry (IHC) analyses.

### RNA sequencing

Total RNA was extracted from rat kidneys in both sham and CS groups at 12 h using RNAiso Plus Total RNA extraction reagent (TAKARA #9109) following the manufacturer’s instructions. RNA integrity was analysed by an Agilent Bioanalyzer 2100 (Agilent technologies). Total RNA was further purified by the RNA Clean XP Kit (Beckman #A63987) and RNase-Free DNase Set (QIAGEN #79254). Shanghai Biotechnology Corporation (Shanghai, China) conducted the RNA library construction and sequencing.

### Serum biochemistry

The blood in both sham and CS groups at 12 h or 36 h was coagulated at 4 °C for 30 min and centrifuged at 3000 r/min for 15 min. The serum was gently collected in a 1.5 ml Eppendorf tube. The levels of creatine kinase (CK), serum creatinine (Scr), blood urea nitrogen (BUN), and myoglobin (Mb) (iMagic, Icubio) were examined by an automatic biochemical analysis instrument (iMagic-V7, Icubio) according to the manufacturer’s instructions. The serum concentrations of IFN-β and TNF-α were determined using a commercial enzyme-linked immunosorbent assay (ELISA) kit (Nanjing Jin Yibai JEB-13712/13718) according to the manufacturer’s protocol.

### Renal histology and immunohistochemistry

The kidney tissues in both sham and CS groups at 12 h or 36 h were washed and fixed in 4% paraformaldehyde for 24 h, dehydrated, and embedded in paraffin (Leica #39601006). Then, the fixed kidney tissues were cut into 4 μm-thick sections and treated with hematoxylin-eosin (HE) and periodic acid-schiff (PAS) stains. Renal tubular injuries were scored by calculating the percentage of tubules that displayed tubular dilation, cast formation and tubular necrosis according to Paller’s method [20]. The sections were also rehydrated and labeled with primary antibodies against RIG-I (1:100, #3743, CST), IRF3 (1:100, #A11373, Affinity), p-IRF3 (1:100, #AF3438, Affinity), p65 (1:100, #AF5006, Affinity), p-p65(ser536) (1:100, #AF2006, Affinity), caspase-3 (1:200, #DF6879, Affinity) and cleaved caspase-3 (1:100, #AF7022, Affinity). Then, the sections were incubated with peroxidase-conjugated secondary antibodies at 37 °C for 1 h. DAB staining was performed, nuclei were counterstained using hematoxylin, and samples were observed by optical microscopy.

### Cell culture and RNA interference

Rat kidney epithelial-like NRK-52E cells (ATCC) were cultured in DMEM-High Glucose (Sparkjade #CA0004) containing 5% fetal bovine serum (Biological Industries #1928703) and 1% Penicillin streptomycin solution (Hyclone #SV30010) at 37 °C and 5% CO_2_. Cells in the exponential growth phase were harvested for experiments. Cells were treated with ferrous myoglobin to mimic the CS-AKI model in vitro [21, 22]. Ascorbic acid (Solarbio #S9490) can reduce myoglobin to a ferrous status [20, 23]. The final concentration of myoglobin was 200 μmol/L while that of ascorbic acid was 2 mmol/L. Small interfering RNAs (siRNAs) were synthesized by GenePharma (China). The sense sequence for siRNA-RIG-I (rat) was 5′-GCCCAUUGAAACCAAGAAAUU-3′ [24]. SiRIG-I was transfected using GP-Transfect-Mate RNAiMAX reagent (GenePharma #201028) following the manufacturer’s protocol. Silencing efficiency of the target gene was verified by PCR and Western blotting analysis.

### Cell viability

NRK-52E cells were cultured in 96 well plates at a concentration of 5 × 10^3^ cells per well and treated with different concentrations of ferrous myoglobin (50, 100, 200, 300, 400, 500, 600, 700, and 800 μmol/L) for 24 h. Then the cells were washed with phosphate-buffered saline (PBS) and cell viability was assessed using a cell counting kit-8 (CCK-8) assay (YEASEN #40203ES60) by measuring absorbance at 450 nm using a Thermo Scientific Microplate Reader.

### Quantitative real-time PCR

For rat kidneys in both groups at 12 h or 36 h and cells samples at 6 h or 24 h, the total RNA was extracted using RNAiso Plus Total RNA extraction reagent (TAKARA #9109). The purity and concentration of RNA were determined using a Nanodrop One. Reverse transcription and qPCR were carried out using a Hifair® III 1st Strand cDNA Synthesis Kit (gDNA digester plus) (YEASEN #11139ES60) and Hieff® qPCR SYBR® Green Master Mix (No Rox) (YEASEN #11201ES03) on a real-time PCR system (LightCycler® 96 Instrument, Roche). The 2^−ΔΔCt^ method was used to calculate the relative expression levels. Primers are listed in Table 1.

**Table 1:** Primer sequences for qPCR

Gene names	Forward primer (5′-3′)	Reverse primer (5′-3′)
Rat KIM-1	GGTCTGTATTGTTGCCGAGTGGAG	GCCTTGTGGTTGTGGGTCTTGTAG
Rat NGAL	CAGGGCAGGTGGTTCGTTGTC	CGAGGATGGAAGTGACGTTGTAGC
Rat RIG-I	GGCTGACTGCTTCCGTTGGTG	CGAGGGCGGCACAGAGTTTG
Rat IRF3	CTTACGACAGGACGCACAGATGG	CAGGTTGACAGGTCTGGCTTATCC
Rat P65	AACACTGCCGAGCTCAAGAT	CATCGGCTTGAGAAAAGGAG
Rat IFN-β	TTGCGTTCCTGCTGTGCTTCTC	TCCGTCCTGTAGCTGAGGTTGAG
Rat TNF-α	AAATGGGCTCCCTCTCATCAGTTC	TCTGCTTGGTGGTTTGCTACGAC
Rat IL-6	ACTTCCAGCCAGTTGCCTTCTTG	TGGTCTGTTGTGGGTGGTATCCTC
Rat caspase-3	AACGGACCTGTGGACCTGAA	TCAATACCGCAGTCCAGCTCT
Rat β-actin	TCACCCACACTGTGCCCATCTATGA	CATCGGAACCGCTCATTGCCGATAG

*KIM-1* Kidney injury molecule-1, *NGAL* Neutrophil gelatinase-associated lipocalin, *RIG-I* Retinoic acid-inducible gene I, *IRF3* Interferon regulatory factor 3, *IFN* interferons, *TNF-α* Tumor necrosis factor-alpha, IL-6: Interleukin-6

### Western blotting

Proteins were extracted from kidney tissue in both groups at 12 h or 36 h and NRK-52E cells at 6 h or 24 h with ice-cold RIPA lysis buffer containing protease inhibitor and phosphatase inhibitor. Samples were centrifuged at 12,000 r/min for 20 min at 4 °C, and the supernatants were immediately collected. Total protein (30 μg) was subjected to SDS-PAGE and subsequently transferred onto polyvinylidene difluoridemembranes. After blocking with 5% skimmed milk for 2 h at room temperature, membranes were incubated overnight with anti-RIG-I (1:1000, #3743, CST), anti-IRF3 (1:1000, #A11373, Affinity), anti-p-IRF3 (1:1000, #AF3438, Affinity), anti-p65 (1:1000, #AF3438, Affinity), anti-p-p65(ser536) (1:1000, #AF3438, Affinity), anti-caspase-3 (1:1000, #AF3438, Affinity), anti-cleaved-caspase-3 (1:1000, #AF3438, Affinity) and anti-GAPDH (1:5000, #KM9002, Sungene Biotech) at 4 °C. The membranes were washed with PBS containing Tween 20 and incubated with HRP-conjugated secondary antibodies (1:5000, #LK2001, Sungene Biotech) for 1 h at room temperature. Protein bands were visualized using ECL chemiluminescence reagent (Tanon #180–501) and imaged by a Tanon 5200 Multi detection system. The intensity of each band was assessed using the Tanon Gel-Pro Analyser system.

### Co-immunoprecipitation

Kidneys from the sham and CS groups at 12 h were lysed using IP lysis buffer (Beyotime #P0013) and incubated with 40 μl protein-A magnetic beads (Bimake #B23201) and 1 μg monoclonal anti-myoglobin antibody at 4 °C under shaking overnight. The magnetic beads were adsorbed with a magnet, and the samples were washed three times with binding buffer and analysed by Western blotting using anti-RIG-I.

### Statistical analysis

Continuous variables with a normal distribution are presented as the mean ± standard deviation (SD). Continuous variables were compared between two groups in siRNA knockdown experiment using the unpaired *t* test, for more groups in the animal experiments using two-way analysis of variance (ANOVA) followed by the Bonferroni’s multiple comparisons test, for more groups in the RIG-I expression by different concentrations of myoglobin or poly (I:C) and the RNA interference experiment using one-way ANOVA followed by the Brown-Forsythe multiple comparisons test. The analyses and figures were implemented with GraphPad Prism 8.0 software. *P*-values below 0.05 were considered to indicate statistical significance.

## Results

### Up-regulated RIG-I expression in kidneys of CS-AKI rats

**Fig. 1:**
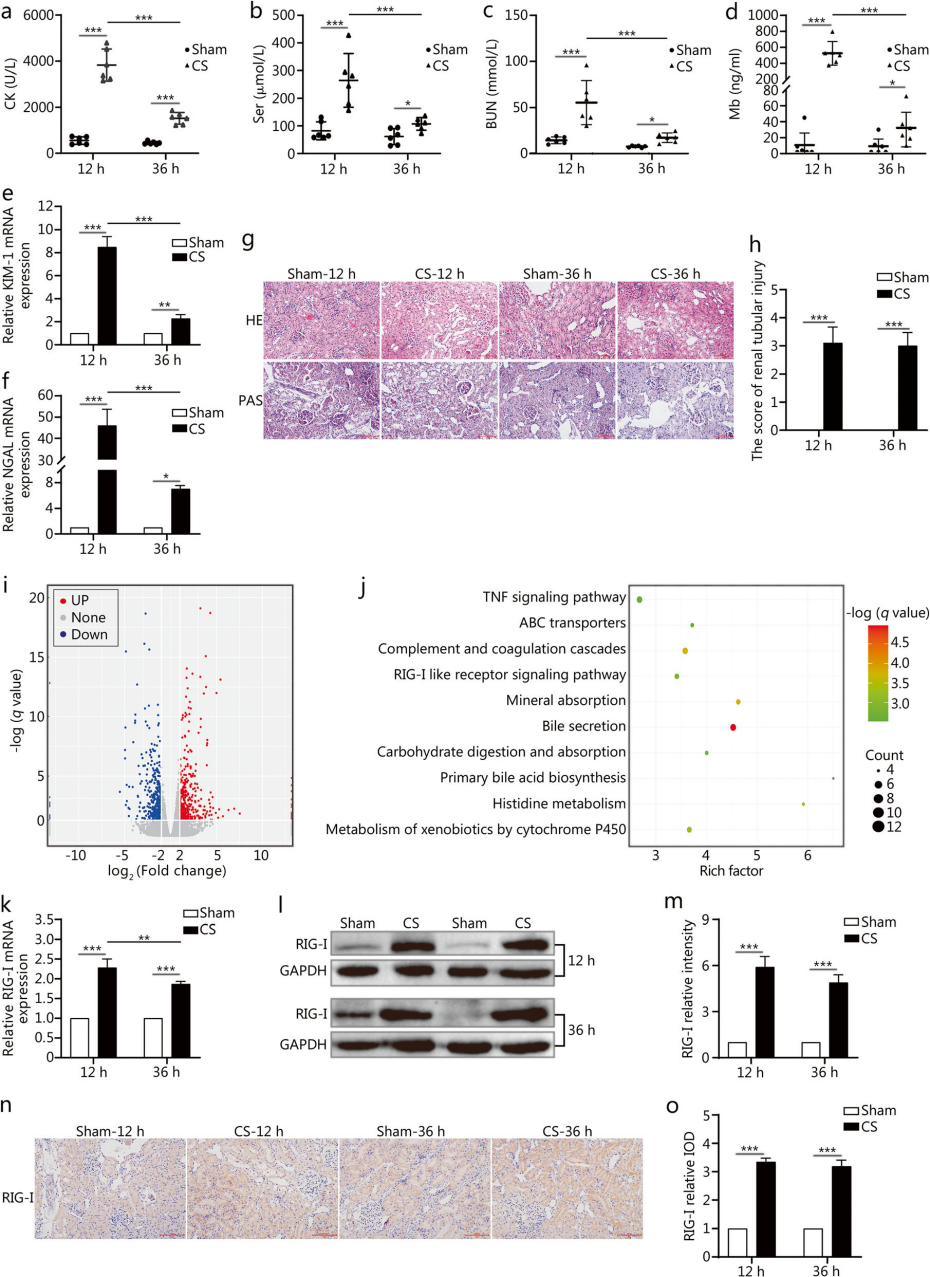
The expression of RIG-I in the CS-AKI rat model. DEGs: Differentially expressed genes. **a–d**. Serum levels of CK, Scr, BUN, and Mb. **e–f**. qPCR analysis of renal KIM-1 and NGAL expression. **g**. HE and PAS staining of kidney tissues (scale bar: 100 μm). **h**. Renal tubular injury score, as determined by calculating the percentage of tubules that displayed tubular dilation, cast formation and tubular necrosis: 0, normal; 1, ≤10%; 2, 10–25%; 3, 26–50%; 4, 51–75%; 5, ≥75%. **i**. DEGs between the sham and CS groups, as identified by RNA sequencing. **j**. The 10 signaling pathways, as identified by KEGG pathway analysis. **k–o**. qPCR, Western blotting and IHC analyses (scale bar: 100 μm) of renal RIG-I expression. Data are expressed as the mean ± SD (*n* = 6). *P < 0.05, **P < 0.01, ***P < 0.001, two-way ANOVA followed by the Bonferroni’s multiple comparisons test

Serum biochemistry analysis results showed that CK levels in the CS group were significantly increased compared with the sham group at12 h (3828.2 ± 692.4 U/L vs. 555.5 ± 164.2 U/L, *P* < 0.001) and 36 h (1520.8 ± 253.9 U/L vs. 451.5 ± 64.1 U/L, *P* = 0.0005) (Fig. 1a). Scr levels were higher in the CS group compared with the sham group at 12 h (264.7 *±* 88.4 μmol/L vs. 82.3 ± 29.6 μmol/L, *P* < 0.001) and 36 h (107.3 ± 20.9 μmol/L vs. 61.3 ± 26.4 μmol/L, *P* < 0.05) (Fig. 1b). The BUN values in the CS group were still higher than those in the sham group at 12 h (55.4 ± 21.9 μmol/L vs. 14.4 ± 3.3 μmol/L, *P* < 0.001) and 36 h (17.2 ± 4.7 μmol/L vs. 7.6 ± 0.9 μmol/L, *P* < 0.05) (Fig. 1c). Meanwhile, the concentration of Mb was also higher in the CS group at 12 h and 36 h (*P* < 0.05, Fig. 1d). Although, the concentrations of CK, Scr, BUN and Mb in the CS group were lower at 36 h compared with 12 h (*P* < 0.001), they were still higher than those in the sham group (Fig. 1a–d). qPCR results showed that renal kidney injury molecule-1 (KIM-1) expression 8.5-fold higher (*P* < 0.001) at 12 h and 2.3-fold higher (*P* = 0.0027) at 36 h (Fig. 1e) in the CS group compared with the sham group. Neutrophil gelatinase-associated lipocalin (NGAL) mRNA expression showed a similar trend (*P* < 0.05, Fig. 1f). Next, we analysed the histological differences between the sham and CS groups through HE and PAS staining. The CS group presented extensive erythrocyte aggregation in the capillaries, congestion, swelling of the glomerulus, and tubular injury (Fig. 1g). The tubular injury score was about 3 points at 12 h and 36 h in the CS group (*P* < 0.001, Fig. 1h).

mRNA was extracted at 12 h from the kidneys of rats from the sham and CS groups, and RNA sequencing was performed. Differentially expressed genes (DEGs) were identified with a threshold of *q* ≤ 0.05 and Fold-change ≥2. A total of 19,800 genes were identified. According to their expression patterns, they were clustered into two groups by hierarchical clustering. Between the sham group and CS group, 749 DEGs were identified (*P* < 0.05), including 386 down-regulated and 363 up-regulated genes (Fig. 1i). Furthermore, kyoto Encyclopedia of Genes and Genomes (KEGG) analysis was performed to identify the main enriched molecular and biological functions of the DEGs. Five classifications were involved in CS-AKI. The top category is organismal systems (Additional Fig. S1a). The number of DEGs involved in the immune system is the highest in the organismal systems (Additional Fig. S1b). KEGG pathway analysis indicated that 170 pathways were involved, and 10 pathways were significantly enriched (*q* < 0.05) (Fig. 1j). Based on the above analysis, the RLR signaling pathway belonged to both the immune system and top 10 pathways. However, the role of the RIG-I signaling pathway in CS-AKI has not been researched.

Therefore, we investigated RIG-I expression in the sham and CS groups. qPCR results showed that renal RIG-I expression was 2.3-fold higher at 12 h and 1.9-fold higher at 36 h (*P* < 0.001, Fig. 1k), while Western blotting analysis revealed that RIG-I expression was 5.9-fold higher at 12 h and 4.9-fold higher at 36 h (*P* < 0.001) in the CS group compared with the sham group (Fig. 1l and m). Tissue IHC analysis confirmed that RIG-I protein expression was higher at 12 h and 36 h in the CS group than those in the sham group (Fig. 1n). Quantification of our IHC results revealed the same changes (*P* < 0.001, Fig. 1o).

### NF-κB/caspase-3 not IRF-3/ IFN-β signaling activated in CS-AKI rat kidney

**Fig. 2:**
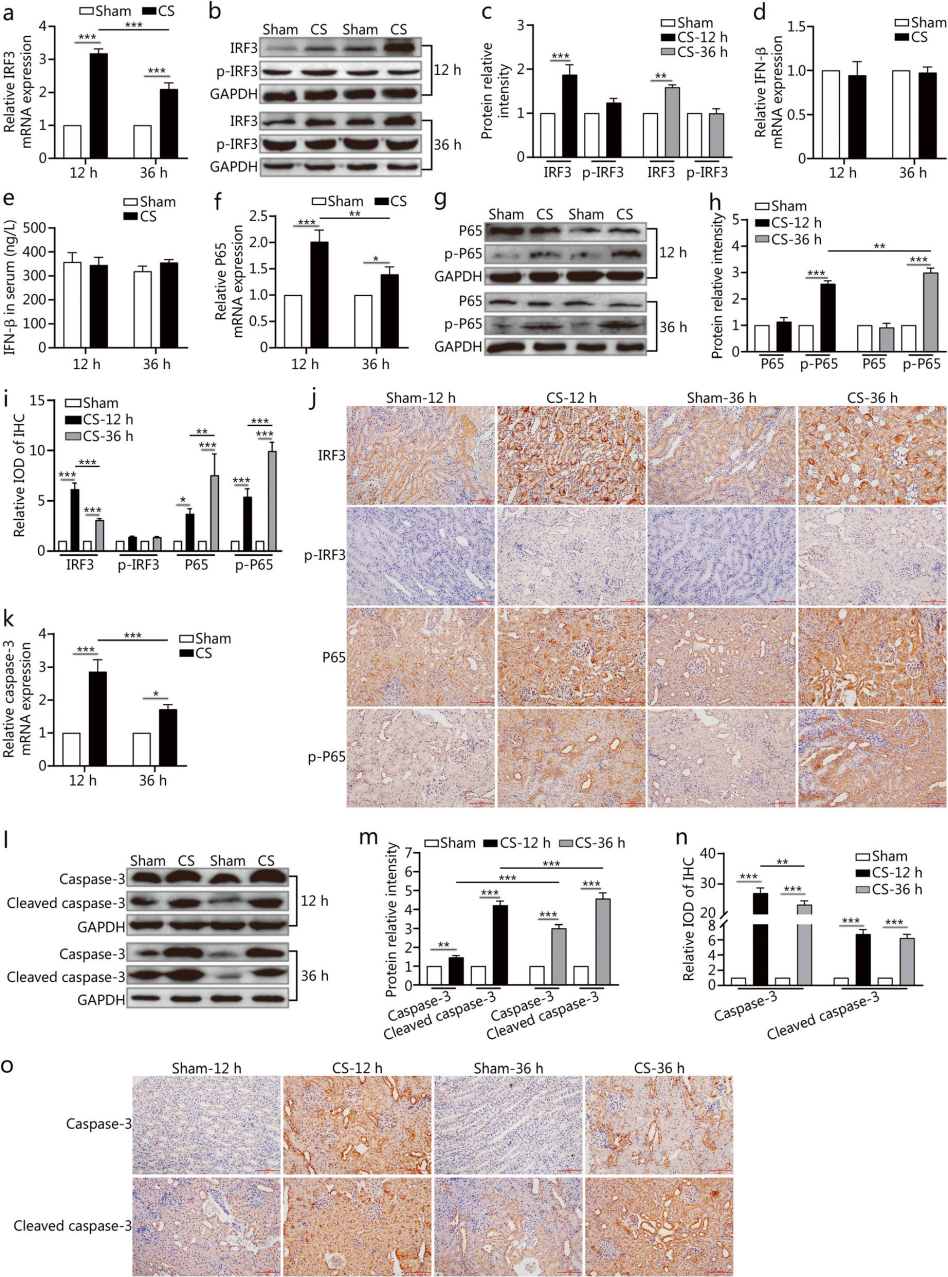
The expressions of downstream molecules involved in RIG-I signaling in the CS-AKI rat model. IRF3: Interferon regulatory factor 3. **a–c**. qPCR, and Western blotting analyses expression levels of renal IRF3. Fig. c is the quantification of Fig. b. **d**. qPCR analyses of renal IFN-β expression. **e**. Serum levels of IFN-β by ELISA. **f–h**. qPCR, and Western blotting analyses renal P65 and p-P65 expression. Fig. h is the quantification of Fig. g. **i–j**. IHC analyses renal IRF3, p-IRF3, P65, and p-P65 expression after relieving the pressure at 12 h and 36 h (scale bar: 100 μm). Fig. i is the quantification of IHC staining. **k–m**. qPCR, and Western blotting analyses renal caspase-3 and cleaved caspase-3 expression. Fig. m is the quantification of Fig. l. **n–o**. IHC analyses renal caspase-3 and cleaved caspase-3 expression (scale bar: 100 μm), Fig. n is the quantification of Fig. o; Data are expressed as the mean ± SD. *P < 0.05, **P < 0.01, ***P < 0.001, two-way ANOVA followed by the Bonferroni’s multiple comparisons test

Next, we investigated which downstream molecules were involved in the RIG-I signaling pathway. Interferon regulatory factor 3 (IRF3) is the classic downstream molecule of RIG-I during viral infection. Hence, we detected the expression of IRF-3 in the kidney. qPCR results indicated that the expression levels of IRF3 were increased at 12 h and 36 h in the kidneys of the CS group compared with the sham group (*P* < 0.001, Fig. 2a). Western blotting results demonstrated that the protein levels of IRF3 were higher at 12 h and 36 h in the kidneys of the CS group than that in the sham group (*P* < 0.01), but renal phospho IRF3 (p-IRF3) levels showed no significant differences between the sham and CS groups at 12 h and 36 h (Fig. 2b and c). qPCR and ELISA results revealed that the expression of IRF3’s effector molecule IFN-β was not significantly different between the sham and CS groups (Fig. 2d and e). Since NF-κB signaling is another downstream branch of RIG-I, we detected the expression of P65, one of the main components of NF-κB. qPCR results indicated that the mRNA levels of P65 were increased at 12 h (*P* < 0.001) and 36 h (*P* = 0.0391) in the kidneys of the CS group (Fig. 2f). Although Western blotting showed that there was no significant difference in P65 protein levels, the levels of the functional form p-P65 were significant increase at 12 h and 36 h in the kidneys of the CS group (*P* < 0.001, Fig. 2g and h). IHC results confirmed that the renal IRF3 expression in the CS group was higher than that in the sham group at 12 h (*P* < 0.001) and at 36 h (*P* < 0.001). Although, p-IRF3 did not show a significant difference, the expression levels of P65 and p-P65 were significantly increased at 12 h and 36 h in the kidney tissue sections of the CS group (*P* < 0.05, Fig. 2i and j). qPCR indicated that the mRNA levels of the pro-inflammatory cytokines interleukin-6 (IL-6) and TNF-α were up-regulated (Additional Fig. S2a and b). ELISA results confirmed that TNF-α levels were increased (Additional Fig. S2b).

Caspase-3 acts as an apoptosis-related protein downstream of the NF-κB signaling pathway. qPCR results indicated that the mRNA expression levels of caspase-3 in the CS group were about 2.9-fold higher at 12 h (*P* < 0.001) and 1.7-fold at 36 h (*P* = 0.0136) than in the sham group (Fig. 2k). Western blotting results indicated that renal caspase-3 and cleaved caspase-3 expression levels were higher in the CS group than those in the sham group (*P* < 0.01, Fig. 2l and m). Meanwhile, tissue IHC results also showed that the protein expression levels of caspase-3 and cleaved caspase-3 were significantly higher in the kidneys of the CS group at 12 h and 36 h (*P* < 0.001, Fig. 2n and o), which is in agreement with the qPCR and Western blotting results.

### RIG-I/NF-κB/caspase-3 axis activated by myoglobin in vitro

**Fig. 3:**
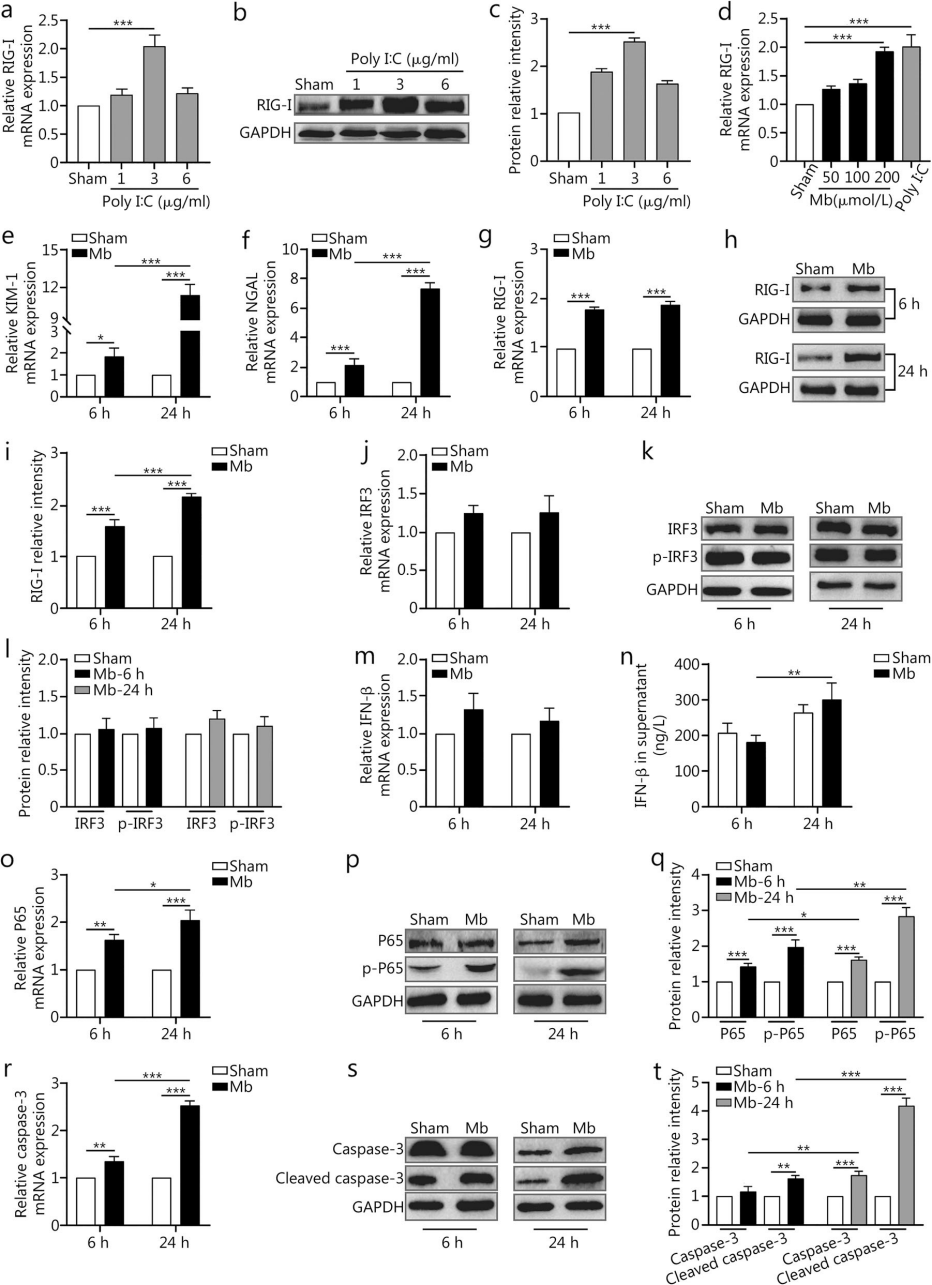
The molecules involved in RIG-I signaling in the CS-AKI cell model. **a–c**. qPCR, and Western blotting analyses the RIG-I expression after NRK-52E cells treatment with 1, 3 or 6 μg/ml Poly I:C for 24 h respectively. Fig. c is the quantification of Fig. b. **d**. qPCR analyses RIG-I expression activated by different concentrations of ferrous myoglobin. **e–f**. qPCR analyses cells KIM-1 and NGAL expression after treatment with 200 μmol/L ferrous myoglobin at 6 h and 24 h separately. **g-i**. qPCR, and Western blotting analyses RIG-I expression. Fig. i is the quantification of Fig. h. **j–l**. qPCR, and Western blotting analyses IRF3 and p-IRF3 expression. Fig. l is the quantification of Fig. k. **m**. qPCR analyses IFN-β expression. **n**. Cell supernatant levels of IFN-β by ELISA. **o–q**. qPCR, and Western blotting analyses P65 and p-P65 expression. Fig. q is the quantification of Fig. p. **r–t**. qPCR, and Western blotting analyses caspase-3 and cleaved caspase-3 expression. Fig. t is the quantification of Fig. s. Data are expressed as the mean ± SD. *P < 0.05, **P < 0.01, ***P < 0.001, two-way ANOVA followed by the Bonferroni’s multiple comparisons test or one-way ANOVA followed by the Brown–Forsythe multiple comparisons test, respectively

Myoglobin is well accepted as the main pathogenic factor for CS-AKI. However, the relationship between myoglobin and RIG-I in CS-AKI is unknown. We used myoglobin to treat rat kidney epithelial-like NRK-52E cells to mimic CS-AKI at the cellular level in vitro to further confirm the molecular mechanisms. Polyinosinic: polycytidylic acid (poly (I:C)), a synthetic RNA analog, could induce RIG-I expression [25]. As shown in Fig. 3a–c, 3 μg/ml poly I:C had the most significant effect on the expression of RIG-I after treating NRK-52E cells for 24 h (*P* < 0.001). The CCK-8 assay results indicated that with increasing myoglobin concentrations, the inhibitory effects on NRK-52E cells increased in a concentration-dependent manner, the IC_50_ was 313 μmol/L (Additional Fig. S3a). Next, we chose different myoglobin concentrations (50, 100 and 200 μmol/L) to treat NRK-52E cells for 24 h. qPCR results indicated that 200 μmol/L ferrous myoglobin was the most suitable dose to activate RIG-I expression, in line with the positive control (*P* < 0.001, Fig. 3d). In addition, qPCR data showed that the mRNA levels of KIM-1 and NGAL strongly increased upon treatment with 200 μmol/L ferrous myoglobin for 6 h (*P* < 0.05) and 24 h (*P* < 0.001) (Fig. 3e and f). As shown in Fig. 3g, when NRK-52E cells were treated with 200 μmol/L ferrous myoglobin, the mRNA expression levels of RIG-I were about 1.8-fold higher (*P* < 0.001) at 6 h and 1.9-fold higher (*P* < 0.001) at 24 h higher than the sham group. Western blotting showed the same trend (*P* < 0.001) (Fig. 3h and i).

Then, we investigated the expression of molecules involved in the RIG-I signaling pathway. After treatment with 200 μmol/L ferrous myoglobin for 6 h and 24 h, qPCR and Western blotting revealed that the expression of IRF3 showed no significant difference (Fig. 3j–l). Moreover, the expression of p-IRF3 did not change significantly compared with the sham group (Fig. 3k–l). qPCR results showed that compared with the sham group, the expression of IFN-β (an effector molecule of IRF3) did not change after treatment with ferrous myoglobin (Fig. 3m). ELISA results indicated that IFN-β expression decreased only a little after treatment with ferrous myoglobin at 6 h, but it did not affect IFN-β expression at 24 h (Fig. 3n). These results basically matched the in vivo results.

Next, we investigated the expression of molecules involved in the RIG-I/NF-κB/caspase-3 signaling pathway. qPCR results showed that the mRNA levels of NF-κB family member P65 were increased about 1.6-fold (*P* = 0.0013) at 6 h and 2.0-fold (*P* < 0.001) at 24 h in the CS group (Fig. 3o). Western blotting showed the protein levels of p65 and p-P65 were also up-regulated after treatment with ferrous myoglobin (*P* < 0.001) (Fig. 3p and q). The mRNA and protein levels of caspase-3 showed the similar trend (*P* < 0.01) (Fig. 3r–t). Protein levels of the functional form of cleaved caspase-3 were significantly up-regulated after incubation with 200 μmol/L ferrous myoglobin for 6 h (*P* = 0.0049) and 24 h (*P* < 0.001) (Fig. 3s and t). qPCR showed that IL-6 and TNF-α were up-regulated in the CS group (*P* < 0.05) (Additional Fig. S3b and c). ELISA results indicated that TNF-α expression was increased in the CS group (Additional Fig. S3d). Pro-inflammatory cytokines were involved in this process.

### RIG-I bound to myoglobin in vivo and knockdown of RIG-I expression alleviate the myoglobin-induced NF-κB /caspase-3 axis activation

**Fig. 4:**
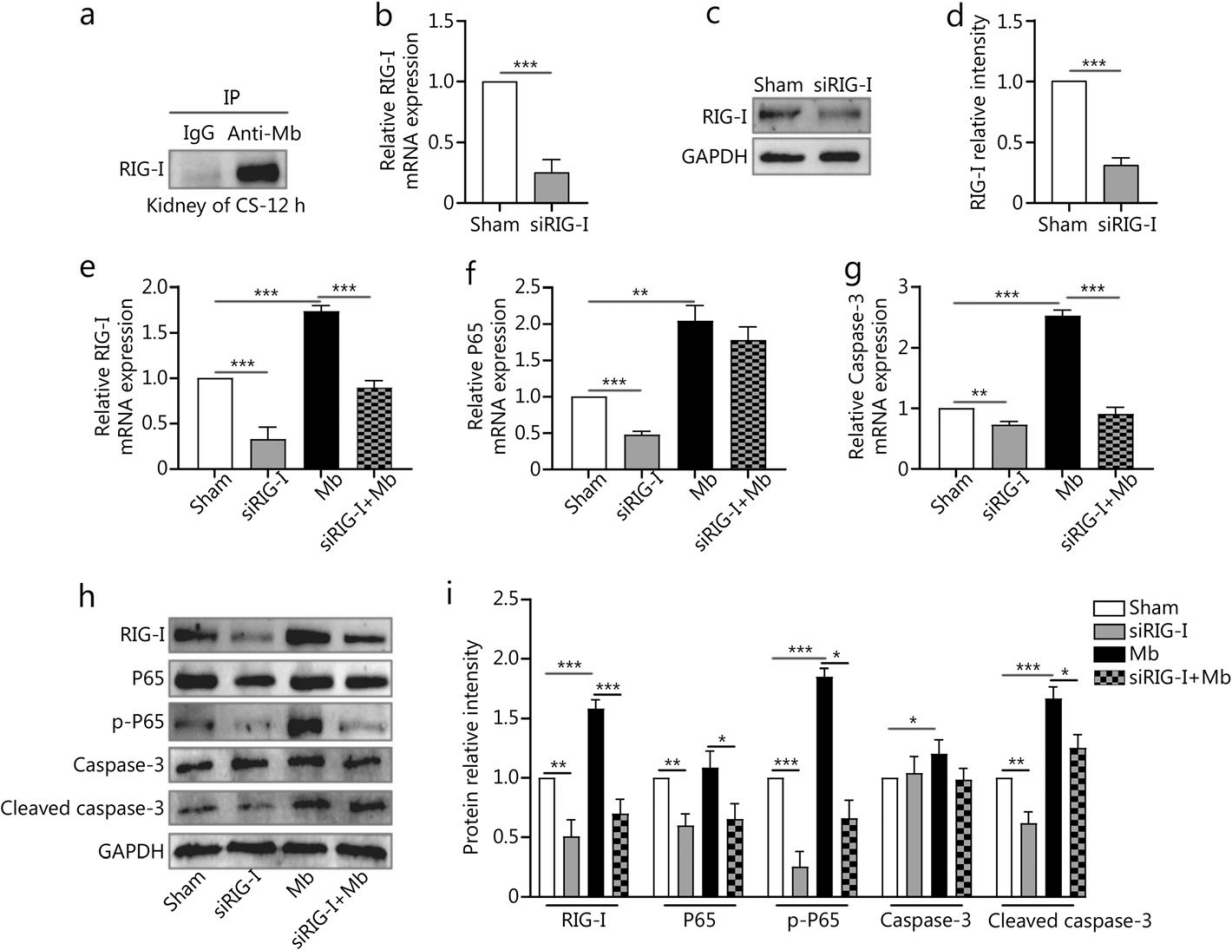
Knocking down RIG-I gene could alleviate the myoglobin induced NF-κB/caspase-3 axis activation. **a**. Co-IP showed the interaction between RIG-I and myoglobin in the rat kidney of the CS group. **b–d**. qPCR, and Western blotting analyses RIG-I expression after NRK-52E cells treatment with siRIG-I. Fig. d is the quantification of Fig. c. **e–i**. qPCR, and Western blotting analyses cells RIG-I, IRF3, p-IRF3, P65, p-P65, caspase-3, and cleaved caspase-3 expression after treatment with ferrous myoglobin and siRIG-I. Fig. i is the quantification of Fig. h. Data are expressed as the mean ± SD. *P < 0.05, **P < 0.01, ***P < 0.001, unpaired *t*-test or by one-way ANOVA followed by the Brown–Forsythe multiple comparisons test, respectively

The co-immunoprecipitation (Co-IP) experiment confirmed that RIG-I binds to myoglobin in the rat kidney of the CS group (Fig. 4a). An RNA interference experiment was performed to investigate the role of RIG-I in a CS-AKI cell model. The semi-quantitative RT-PCR, qPCR, and Western blotting results showed that the knockdown efficiency was about 70% (*P* < 0.001) (Fig. 4b–d, Additional Fig. S4a). After depletion of RIG-I, the mRNA levels of P65 (*P* < 0.001) and caspase-3 (*P* < 0.01) were decreased (Fig. 4f-i). The protein levels of p-P65 (*P* < 0.001) and cleaved caspase-3 (active forms) (*P* < 0.01) were also decreased (Fig. 4h and i). qPCR results showed that knockdown of RIG-I alleviated the effects of myoglobin on the expression of RIG-I and caspase-3 at mRNA level (*P* < 0.001, Fig. 4e and g). Western blotting showed that the protein expression levels of RIG-I (*P* < 0.001), p-P65 (*P* < 0.05), and cleaved caspase-3 (*P* < 0.05) showed a similar trend (Fig. 4h and i). Furthermore, qPCR showed that the expression of IL-6 and TNF-α were in good agreement with the above results (Additional Fig. S4b and c). The protein level of TNF-α showed the same trend (Additional Fig. S4d).

## Discussion

Myoglobin acts as a DAMPs and is the primary cause of CS-AKI. The pathogenic mechanism of CS is similar to that of ordinary rhabdomyolysis [26, 27]. Myoglobin triggers multiple nephrotoxic pathways by tubular precipitation and obstruction, vasoconstriction of renal afferent arterioles, and oxidative stress at the tubular level [28, 29]. Unfortunately, the exact mechanisms underlying the toxicity of myoglobin to renal cells are not clear in CS-AKI (or RM-AKI). Thus, we performed in vivo and in vitro experiments to clarify the mechanisms of underlying CS-AKI. RNA sequencing analysis revealed differentially expressed genes involved in the RLR signaling pathway between the sham and CS groups, shedding light on the role of the RIG-I/NF-κB/caspase-3 signaling pathway in the CS-AKI model. Therefore, RIG-I is a novel sensor for myoglobin in the CS-AKI model, and myoglobin-induced RIG-I/NF-κB/caspase-3 axis activation may contribute to the development of CS or rhabdomyolysis nephrotoxicity.

Usually, KIM-1, NGAL, and CK are used to assess the degree of kidney damage [30, 31] and the prognosis of AKI [32–34]. Myoglobin has a weaker positive correlation with the severity of CS and the prognosis of AKI compared with the level of CK (5-fold higher than normal or CK cut-off value more than 1000 U/L) in clinical applications [32–34]. In the present study, the serum level of myoglobin was still significantly increased in the CS-AKI rat model.

Based on our previous research, the state of CS rats is the most severe at 12 h after decompression [18, 19]. Therefore, we chose the time point at 12 h for RNA sequencing analysis. KEGG pathway analysis indicated that DEGs were highly enriched in 10 pathways, such as the RLR signaling pathway. Most studies on RIG-I focus on viral infections, and there are few related studies to explore the role of RIG-I in traumatic AKI. Our RNA sequencing results indicate the role of RIG-I in CS-AKI.

Previous research indicated that RIG-I activates IRF3 through the RIG-I/MAVS/TRAF3/TBK-1 signaling pathway, and then induces the production of IFNs to perform antiviral functions. RIG-I can also activate the NF-κB signaling pathway through the RIG-I/MAVS/CARD/Bcl-10 pathway, to induce apoptosis and eliminate viral particles. However, it is unknown which signaling pathway plays a major role in CS-AKI. Our research indicated that RIG-I/NF-κB/caspase-3 is the main activation pathway, while the classic virus-activated RIG-I/IRF3/IFN-β signaling pathway was not activated in CS-AKI. IL-6 and TNF-α are considered to be inflammation mediators in various injuries [35, 36], and have adverse impacts on the development of CS-AKI. The levels of TNF-α and IL-6 were notably elevated in the CS group.

**Fig. 5:**
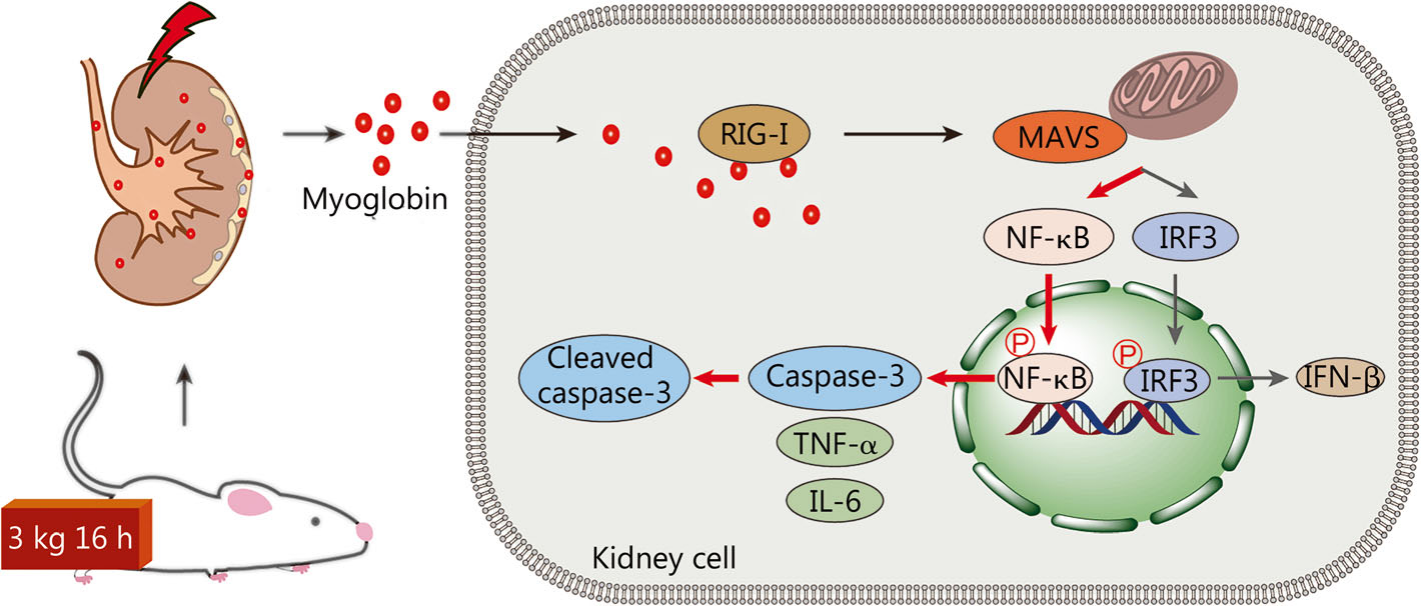
Schematic drawing of the novel RIG-I signaling pathway in CS-AKI. In the crush syndrome related acute kidney injury (CS-AKI) rat model, muscle tissue releases large amounts of myoglobin to injure the kidney. Myoglobin binds to RIG-I after endocytosis, then mainly activate NK-κB/caspase-3 axis, not the classical IRF-3/IFN-β signaling pathway in CS-AKI rat kidney. In this process, the expression of pro-inflammation cytokines IL-6 and TNF-α are also increased to some degree

To underpin the above finding, we incubated NRK-52E cells with ferrous myoglobin to mimic CS-AKI at the cellular level in vitro [21, 37]. After treatment with 200 μmol/L ferrous myoglobin, the mRNA and protein levels of RIG-I, P65, p-P65, caspase-3 and cleaved caspase-3 were increased. The expression decreased after knockdown of RIG-I in NRK-52E cells. Because negative control siRNA can act as exogenous RNA to influence the expression of RIG-I [38], we did not treat control cells with negative control siRNA, but used normal NRK-52E cells as the control group. The results indicated that myoglobin upregulates the levels of the apoptosis-related proteins NF-κB and caspase-3. We thus show that RIG-I is a novel DAMPs sensor (recognizing myoglobin) in CS-AKI, and myoglobin can activate the RIG-I/NF-κB/caspase-3 signaling pathway. The functional mechanism of RIG-I in CS-AKI differs from the classical innate immunity activated by viral nucleic acid (Fig. 5).

Recent research indicated that in the absence of viral infection, RIG-I could also be activated by a pattern recognition receptor in kidney-related inflammatory diseases [14, 39, 40]. Simultaneously, the relationship between RIG-I and traumatic diseases has been reported. de Rivero Vaccari JP et al. [17] revealed that spinal cord injury promotes gliosis via an RIG-like signaling pathway. Fischer et al. [15] suggested that RIG-I signaling rescues gut integrity in irradiation- and immune-mediated intestinal tissue injury. However, there is no research about the role of RIG-I signaling pathway in traumatic kidney injury. RIG-I is ubiquitously expressed in epithelial cells and myeloid cells [41] and our results revealed that myoglobin, after absorption by renal tubular epithelial cells could induce RIG-I activation. Therefore, our results suggest that RIG-I is a potential therapeutic target for CS-AKI.

The present work has limitations. We need to explore in-depth how myoglobin triggers the cytoplasmic pattern recognition receptor RIG-I. We need additional animal studies to confirm the therapeutic effect of RIG-I in CS-AKI, for example using RIG-I knockout mice. In addition, clinical studies need to be performed to confirm the role of RIG-I in patient samples.

## Conclusions

In summary, our results demonstrate that RIG-I is activated upon interaction with myoglobin in the CS-AKI model. Knockdown of RIG-I in a CS-AKI cell model attenuated myoglobin toxicity in NRK-52E cells through the NK-κB/caspase-3 axis. This is the first investigation of the relationship between CS and the RIG-I signaling pathway, and our results indicate that RIG-I is a novel DAMPs sensor, recognizing myoglobin and activating the NK-κB/caspase-3 axis in CS-AKI (Fig. 5). Our study suggested that RIG-I may serve as a therapeutic target for the prevention or treatment of CS-AKI or RM-AKI.

## Supplementary Information

**Additional file 1: Fig. S1**. KEGG analysis identifies the main enriched molecular and biological functions of the DEGs. a. KEGG classification analyses DEGs between the sham and CS groups. The involved five classifications are organismal systems, environmental information processing, metabolism, cellular processes and genetic information processing. b. The detail information of organismal systems.

**Additional file 2: Fig. S2**. The expressions of inflammation mediators involved in RIG-I signaling in CS-AKI rat model. a–b. qPCR analyses the renal IL-6 and TNF-αexpression after relieving the pressure at 12 h and 36 h. c. Serum levels of TNF-α by ELISA after relieving the pressure at 12 h and 36 h between the sham and CS groups. Data are expressed as the mean ± SD. *P < 0.05, **P < 0.01, ***P < 0.001, two-way ANOVA followed by the Bonferroni’s multiple comparisons test.

**Additional file 3: Fig. S3**. The inhibitory effects on NRK-52E cells and inflammation meditators expression between the sham and ferrous myoglobin treatment groups. a. CCK8 assays analyses the inhibitor effects on NEK-52E cells after treatment with ferrous myoglobin. The ferrous myoglobin concentration is 50, 100, 200, 300, 400, 500, 600, 700, 800 μmol/L. b-c. qPCR analyses IL-6 and TNF-α expression in the NRK-52E cells after treatment with 200 μmol/L ferrous myoglobin at 6 h and 24 h separately. d. Cell supernatant levels of TNF-α by ELISA between the sham and ferrous myoglobin treatment groups. Data are expressed as the mean ± SD. *P < 0.05, **P < 0.01, ***P < 0.001, two-way ANOVA followed by the Bonferroni’s multiple comparisons test.

**Additional file 4: Fig. S4**. Inflammation meditators expression after knockdown RIG-I gene in NRK-52E cells. a. Agarose gel verified the knockdown efficiency after siRIG-I treatment. b-c. NRK-52E cells treatment with 200 μmol/L ferrous myoglobin at 6 h, or using siRNA to knockdown RIG-I gene before treatment with ferrous myoglobin. qPCR analyses the IL-6 and TNF-α expression. d. Cells supernatant levels of TNF-α by ELISA after different treatment. Data are expressed as the mean ± SD. *P < 0.05, **P < 0.01, ***P < 0.001, one-way ANOVA followed by the Brown-Forsythe multiple comparisons test.

## Data Availability

The data and materials used in the current study are all available from the corresponding author upon reasonable request.

## Abbreviations

AKI: Acute kidney injury
BUN: Blood urea nitrogen
CCK-8: Cell counting kit 8
CK: Creatine kinase
CKD: Chronic kidney disease
CS: Crush syndrome
DAMPs: Damage-associated molecular patterns
DEGs: Differentially expressed genes
ELISA: Enzyme-linked immunosorbent assay
HE: Hematoxylin-eosin
IFN: Interferons
IHC: Immunohistochemistry
IL-6: Interleukin-6
IRF3: Interferon regulatory factor 3
KIM-1: Kidney injury molecule-1
KEGG: Keto encyclopedia of genes and genomes
Mb: Myoglobin
NGAL: Neutrophil gelatinase-associated lipocalin
PAMPs: Pathogen-associated molecular patterns
PAS: Periodic acid-schif
Poly(I:C): Polyinosinic:polycytidylic acid
PVDF: Polyvinylidene difluoride
PS: Penicillin-Streptomycin Solution
qPCR: Quantitative Real-time PCR
RIG-I: Retinoic acid-inducible gene I
RLRs: RIG-I-like receptors
RM-AKI: Rhabdomyolysis syndrome related AKI
Scr: Serum creatinine
SD: Sprague-Dawley
TNF-α: Tumor necrosis factor-alpha
