# Supplementary figures and images for: RIG-I, a novel DAMPs sensor for myoglobin activates NF-κB/caspase-3 signaling in CS-AKI model
Source: Mil Med Res. 2021 Jun 21;8:37. doi: 10.1186/s40779-021-00333-4 (PMC8215750; doi:10.1186/s40779-021-00333-4)

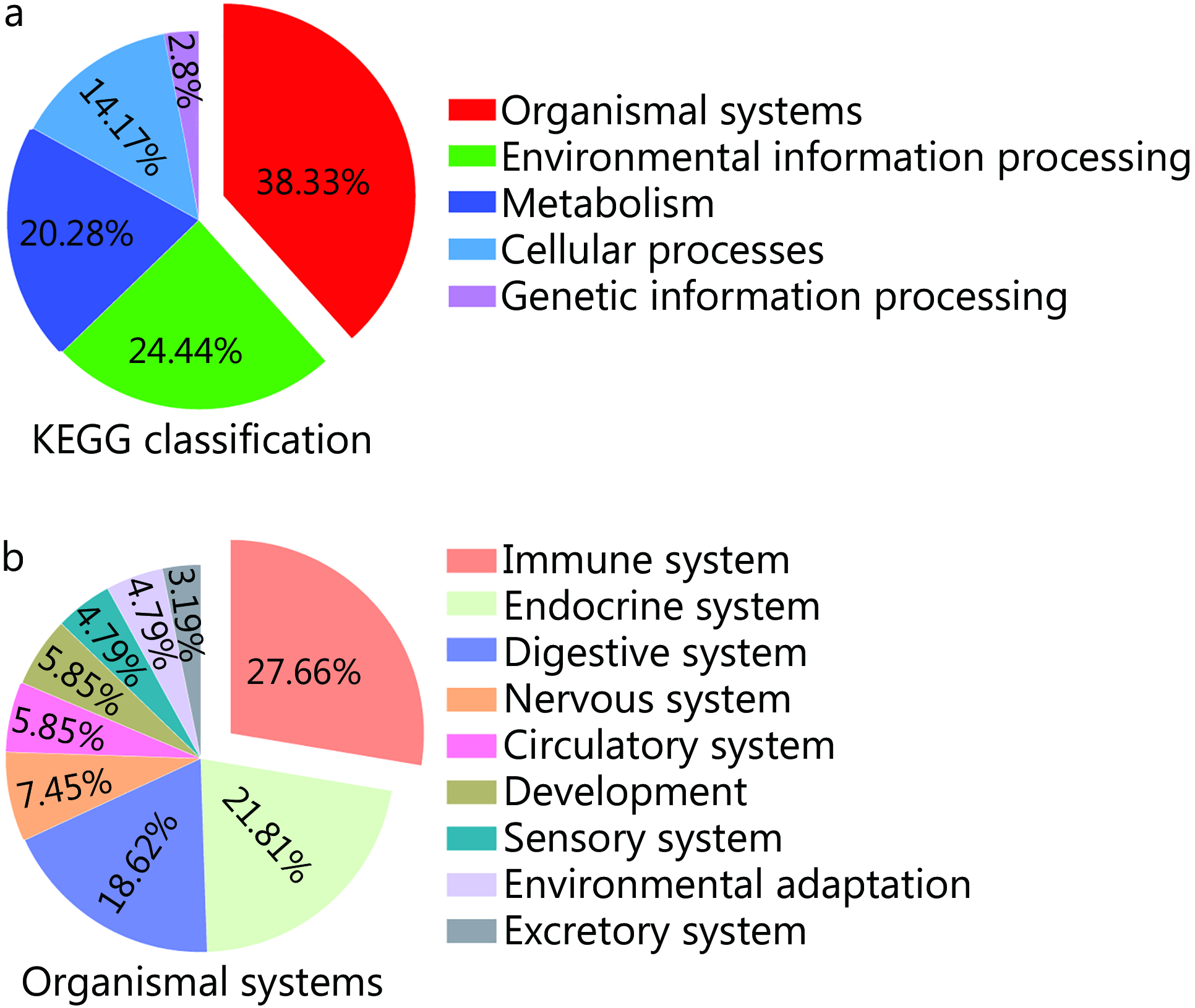

Supplement: Supplementary file 1 — Additional file 1: Fig. S1. KEGG analysis identifies the main enriched molecular and biological functions of the DEGs. a. KEGG classification analyses DEGs between the sham and CS groups. The involved five classifications are organismal systems, environmental information processing, metabolism, cellular processes and genetic information processing. b. The detail information of organismal systems. [file 40779_2021_333_MOESM1_ESM.tif]

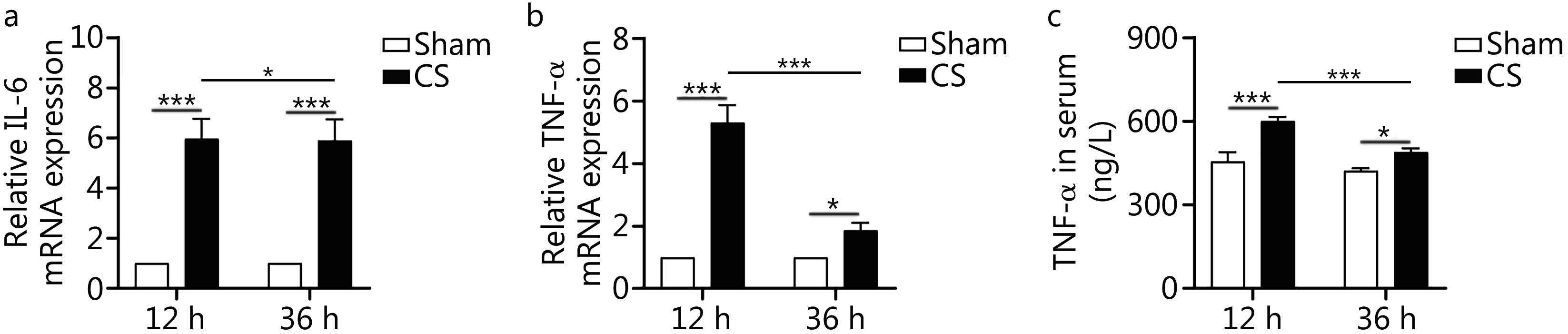

Supplement: Supplementary file 2 — Additional file 2: Fig. S2. The expressions of inflammation mediators involved in RIG-I signaling in CS-AKI rat model. a–b. qPCR analyses the renal IL-6 and TNF-αexpression after relieving the pressure at 12 h and 36 h. c. Serum levels of TNF-α by ELISA after relieving the pressure at 12 h and 36 h between the sham and CS groups. Data are expressed as the mean ± SD. *P < 0.05, **P < 0.01, ***P < 0.001, two-way ANOVA followed by the Bonferroni’s multiple comparisons test. [file 40779_2021_333_MOESM2_ESM.tif]

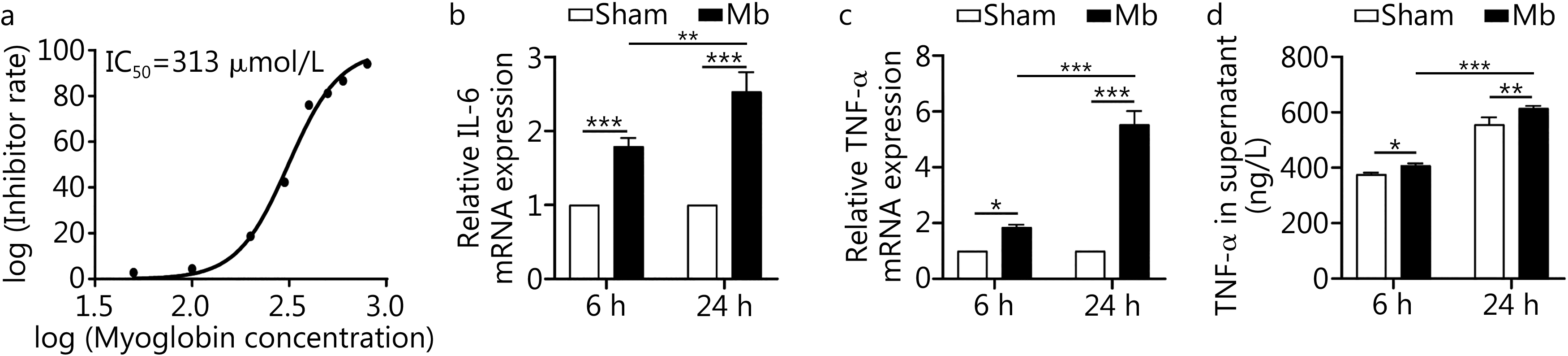

Supplement: Supplementary file 3 — Additional file 3: Fig. S3. The inhibitory effects on NRK-52E cells and inflammation meditators expression between the sham and ferrous myoglobin treatment groups. a. CCK8 assays analyses the inhibitor effects on NEK-52E cells after treatment with ferrous myoglobin. The ferrous myoglobin concentration is 50, 100, 200, 300, 400, 500, 600, 700, 800 μmol/L. b-c. qPCR analyses IL-6 and TNF-α expression in the NRK-52E cells after treatment with 200 μmol/L ferrous myoglobin at 6 h and 24 h separately. d. Cell supernatant levels of TNF-α by ELISA between the sham and ferrous myoglobin treatment groups. Data are expressed as the mean ± SD. *P < 0.05, **P < 0.01, ***P < 0.001, two-way ANOVA followed by the Bonferroni’s multiple comparisons test. [file 40779_2021_333_MOESM3_ESM.tif]

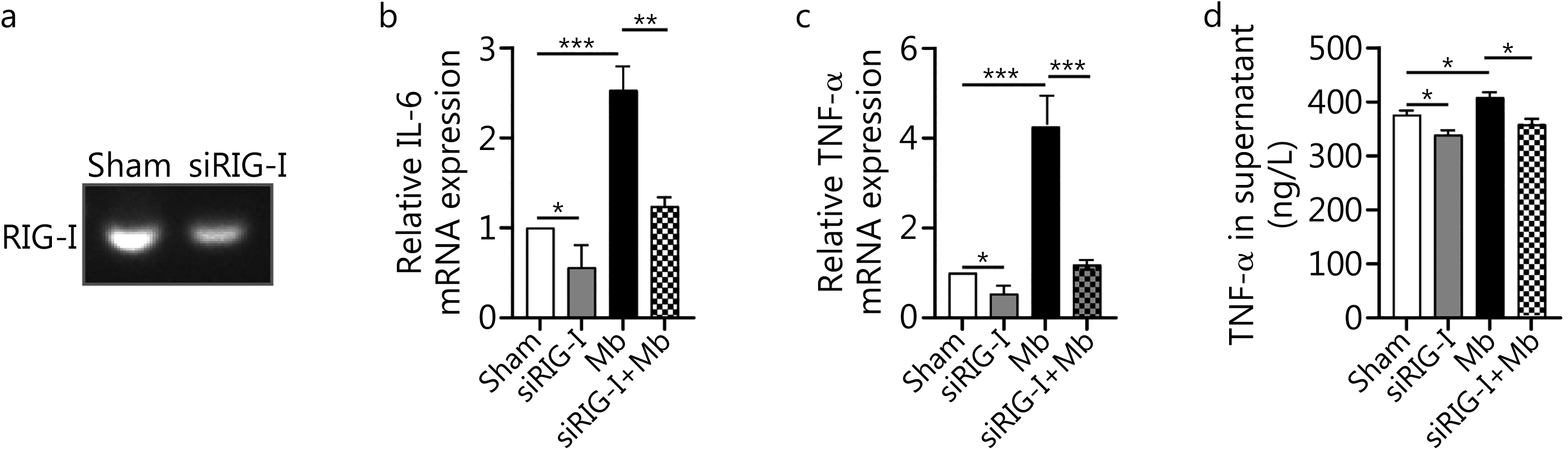

Supplement: Supplementary file 4 — Additional file 4: Fig. S4. Inflammation meditators expression after knockdown RIG-I gene in NRK-52E cells. a. Agarose gel verified the knockdown efficiency after siRIG-I treatment. b-c. NRK-52E cells treatment with 200 μmol/L ferrous myoglobin at 6 h, or using siRNA to knockdown RIG-I gene before treatment with ferrous myoglobin. qPCR analyses the IL-6 and TNF-α expression. d. Cells supernatant levels of TNF-α by ELISA after different treatment. Data are expressed as the mean ± SD. *P < 0.05, **P < 0.01, ***P < 0.001, one-way ANOVA followed by the Brown-Forsythe multiple comparisons test. [file 40779_2021_333_MOESM4_ESM.tif]
